# Synchronized multi-wavelength soliton fiber laser via intracavity group delay modulation

**DOI:** 10.1038/s41467-021-26872-x

**Published:** 2021-11-18

**Authors:** Dong Mao, Huaqiang Wang, Heze Zhang, Chao Zeng, Yueqing Du, Zhiwen He, Zhipei Sun, Jianlin Zhao

**Affiliations:** 1 MOE Key Laboratory of Material Physics and Chemistry Under Extraordinary Conditions, and Shaanxi Key Laboratory of Optical Information Technology, School of Physical Science and Technology, Northwestern Polytechnical University, Xi’an, 710129 China; 2 Department of Electronics and Nanoengineering and QTF Centre of Excellence, Aalto University, Aalto, Finland

**Keywords:** Solitons, Ultrafast photonics

## Abstract

Locking of longitudinal modes in laser cavities is the common path to generate ultrashort pulses. In traditional multi-wavelength mode-locked lasers, the group velocities rely on lasing wavelengths due to the chromatic dispersion, yielding multiple trains of independently evolved pulses. Here, we show that mode-locked solitons at different wavelengths can be synchronized inside the cavity by engineering the intracavity group delay with a programmable pulse shaper. Frequency-resolved measurements fully retrieve the fine temporal structure of pulses, validating the direct generation of synchronized ultrafast lasers from two to five wavelengths with sub-pulse repetition-rate up to ~1.26 THz. Simulation results well reproduce and interpret the key experimental phenomena, and indicate that the saturable absorption effect automatically synchronize multi-wavelength solitons in despite of the small residual group delay difference. These results demonstrate an effective approach to create synchronized complex-structure solitons, and offer an effective platform to study the evolution dynamics of nonlinear wavepackets.

## Abstract (web summary)

The coherence degradation of pulses synchronized to optical cavities is an issue for ultrahigh-repetition-rate lasing. Here the authors demonstrate synchronized multi-wavelength mode-locked soliton fiber lasers generating ultrafast outputs from two to five wavelengths with a high sub-pulse repetition rate.

## Introduction

The modes of a laser are a group of stationary waves that satisfy the resonant condition of the laser cavity. In general, they are three-dimensional optical fields varying along the resonator, manifested as longitudinal modes and transversal modes in the frequency and spatial domains, respectively^1^. In the past decades, many techniques have been developed to manipulate the longitudinal modes of lasers. For example, single-longitudinal-mode fiber lasers have been demonstrated with ultra-narrow spectral filters^2^ and multi-ring cavity configurations^3^. Assisted by ultrafast saturable absorbers, a large number of longitudinal modes can be passively locked inside cavities, generating nanosecond^4^ to femtosecond laser pulses^5–7^. Based on a nonlinear microcavity, couples of longitudinal modes were filtered out from a fiber laser and then locked via the four-wave mixing effect^8^. Beside these, mode-locked fiber lasers can also be actively controlled via programmable spectral filters, capable of improving pulse parameters^9^, and generating multi-type solitons^10^ as well as pure-quartic solitons^11^.

Recently, by leveraging the birefringence-induced filter and the double-hump gain spectrum, dual-wavelength mode-locked solitons have been demonstrated with ytterbium-^12^, erbium-^13,14^, and thulium-doped fiber lasers^15^. Affected by the group delay dispersion (GDD), such dual-wavelength solitons have different group velocities, and thus collide periodically inside the laser cavity^16^. To synchronize dual-wavelength pulses, the laser cavity dispersion should confine to a small range. For solid-state lasers, unsynchronized^17^ and synchronized^18–20^ dual-wavelength mode-locking states have been realized with two-peak gain media. Similar operations can also be achieved in fiber lasers by trimming the cavity dispersion to near-zero regime^21^ or introducing birefringence-related filtering effect^22^. Due to the gain competition effect, the aforementioned lasers are difficult to support multi-wavelength synchronized pulses (i.e., ≥three wavelengths). Although two pulses at different wavelengths can also be synchronized external to cavities based on active^23,24^ or passive^25,26^ methods, the coherence and stability degrade as they experience different physical paths and environmental disturbances during propagation^27,28^. As such, the very attractive topic of ultrafast lasers is to synchronize multi-wavelength solitons in non-zero-dispersion cavities, which are highly desirable for applications of field-enhancement Raman microscopy^29,30^, difference-frequency terahertz-wave generation^31^, and drift-free timing synchronization^24^.

Here, we demonstrate synchronized multi-wavelength mode-locked soliton fiber lasers by imparting the group delay compensation and spectral filtering effects with a programmable pulse shaper (PPS), enabling the direct generation of two- to five-wavelength solitons with sub-pulses repetition rates of 0.4–1.26 THz. Numerical simulations fully interpret experimental observations, and indicate that the saturable absorption effect automatically arranges multiple spectra into a synchronized state in despite of the small residual group delay difference.

## Results

### Principle and setup

**Fig. 1:**
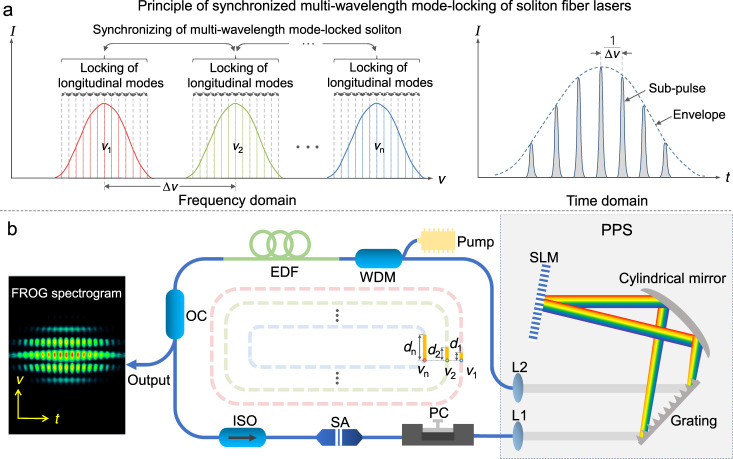
Synchronized multi-wavelength mode-locking of soliton pulses. **a** Operation principle of synchronized multi-wavelength soliton fiber laser in frequency and time domain. **b** Sketch of the mode-locked fiber laser. WDM wavelength division multiplexer, EDF erbium-doped fiber, OC optical coupler, PI-ISO polarization insensitive isolator, SA saturable absorber, PC polarization controller, PPS programmable pulse shaper, SLM spatial light modulator, L1–L2 collimating lenses, FROG frequency-resolved optical gating. The group delay difference (*d*_1_ to *d*_*n*_) among the multiple spectra (*ν*_1_ to *ν*_*n*_) can be counteracted by introducing group delay with the SLM. The inset shows the typical FROG spectrogram of synchronized dual-wavelength mode-locked fiber lasers.

Figure 1 shows the concept and experiment setup of the synchronized multi-wavelength mode-locked soliton fiber lasers via intracavity group delay modulation. The longitudinal modes of a typical mode-locked fiber laser have a constant phase difference and interfere constructively at the in-phase points, forming a train of ultrashort pulses in the time domain^32^. For conventional multi-wavelength mode-locked lasers, the pulse velocities depend on the operating wavelengths due to the intracavity GDD. However, when the GDD is negligible or the group delay difference is compensated, the pulses centered at different wavelength can be synchronized, generating a packet of ultrahigh-repetition-rate pulses (Fig. 1a). Figure 1b shows the configuration of the fiber laser, where the PPS adjusts the group delay as well as the amplitude of each spectral component and the carbon nanotube saturable absorber (CNT-SA) achieves passive mode-locking. The total optical length and net dispersion of the cavity are 18.9 m and −0.52 ps^2^, respectively.

### Synchronized dual-wavelength mode-locking of soliton fiber laser

**Fig. 2:**
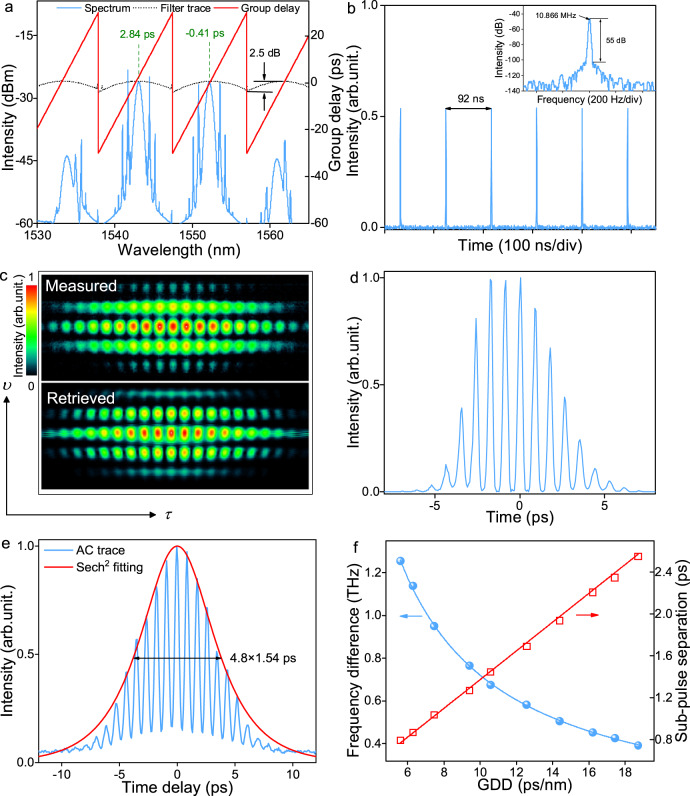
Experiment results of synchronized dual-wavelength mode-locked solitons. **a** Spectrum of pulses, filtering and group delay imparted by the PPS. The imparted group delay difference between two spectra is 3.25 ps, for partially compensating that induced by the intrinsic dispersion of PPS and cavity. **b** Pulse train and radio frequency spectrum of the wavepacket. **c** Measured (upper panel) and retrieved (lower panel) FROG spectrograms. **d** Retrieved pulse profile. **e** Autocorrelation (AC) trace and the Sech^2^ fitting. **f** Frequency difference and sub-pulse separation versus imparted GDD.

The fiber laser operates at a stable single-wavelength mode-locked state before imparting group delay with the PPS, where conventional solitons are formed with the intrinsic balance between the anomalous group-velocity dispersion and self-phase modulation effect^33^, as illustrated in Supplementary Note 1. After introducing the zigzag group delay, the transmission of PPS varies correspondingly with the wavelength due to the amplitude and phase modulation realized via the first order approach^34^, as illustrated by the black and red curves in Fig. 2a. One can observe that, the transmission approaches to maximum at the zero-group-delay wavelength, while reduces quadratically when the group delay deviates from this value. Thus, a periodic spectral filter with the modulation depth of ~2.5 dB always accompanies with the zigzag group delay, which can roughly confine the locations of spectra. It is worth to mention that the modulation depth of the filter decreases significantly with the amplitude of group delay function.

Self-starting synchronized dual-wavelength mode-locking state can be established in the fiber laser at the pump power of 22 mW when the PPS-induced GDD spans from 5.6 to 18.7 ps/nm. Figure 2a–d shows a typical synchronized dual-wavelength solitons centered at 1543.1 and 1552.2 nm for the GDD of 6.3 ps/nm. The group delays at two peak wavelengths imparted by the PPS are 2.84 and −0.41 ps, giving the group delay difference of 3.25 ps. Thus, after inserting the PPS into cavity, the net group delay difference between two wavelengths diminishes from 3.68 to 0.43 ps. Two completely separated spectra, with pairs of soliton sidebands, have the similar profile with the same bandwidth of 0.66 nm, validating that the fiber laser operates at the anomalous-dispersion regime^6,7^. Two weaker attendant spectra at 1533.8 and 1560.9 nm typically accompany with the stronger spectra (with intensity difference of ~17 dB), which can be attributed to the four-wave mixing effect as their powers are far less than the formation threshold of solitons. The slight asymmetry of the spectral sidebands may result from third-order and other high-order dispersion of the fiber laser^35^.

Compared with unsynchronized dual-wavelength soliton lasers where two pulses circulate independently per roundtrip, only one pulse can be observed on the oscilloscope in our experiment, as shown in Fig. 2b. The radio frequency spectrum at the span of 1 kHz and the resolution of 9.1 Hz exhibits a single-peak structure with a signal-to-noise ratio higher than 55 dB (inset of Fig. 2b). At the 1 GHz span, there is no other strong intensity modulation on the radio frequency spectrum, indicating the good stability of the synchronized dual-wavelength operation (Supplementary Fig. 2a). The fine structure of pulses can be retrieved with frequency-resolved optical gating (FROG) technique^36^ or spectral interferometry for direct electric field reconstruction measurement^37^. Based on a second-harmonic generation FROG, we perform a set of spectral-resolved measurements to gain insight into the temporal properties of the pulses (Fig. 2c, d). The retrieved FROG spectrogram coincides with that of the measured counterpart, confirming the reliability of the spectral-resolved measurements. Note that the retrieved pulse displays a stable multi-peak temporal structure, indicating that the two pulses are synchronized in the time domain. Here, the separation of neighboring sub-pulses is 0.87 ps, corresponding to the sub-pulse repetition rate of 1.14 THz. The pulse envelope has a full-width at half-maximum of ~4.8 ps using the Sech^2^ fitting, as endorsed by the autocorrelation trace in Fig. 2e. At the maximum span of 150 ps, only single wavepacket is observed on the autocorrelator, as shown in Supplementary Fig. 2b.

The time-bandwidth product of the envelope is ~0.4, confirming the formation of near-Fourier-transform-limited pulses. In most states, the two spectra have the same polarization state, however, at certain polarization controller settings, the orthogonally-polarized operations are also observed and the weaker attendant spectra vanish, as elaborated in Supplementary Note 3. But, the orthogonally polarized operation state is less table and can easily transform to the same polarization operation state with minor environmental disturbance mainly because the saturable absorber at the same polarization operation state has a higher instantaneous transmittance than that at the orthogonally polarized operation state.

We further study the synchronized dual-wavelength mode-locked solitons by filtering out each spectrum external to the cavity. The constituent pulses at two different wavelengths have comparable durations as that of the pulse envelope (Supplementary Fig. 4), consistent with the Fourier transform principle^38^ and mode-locking theory^1^. The performance of dual-wavelength synchronized mode-locked solitons depends on the GDD and bandwidth introduced by the PPS. As described in Fig. 2f, the frequency difference of two mode-locked spectra, equivalent to the sub-pulses repetition rate, decreases from 1.26 to 0.4 THz by increasing the GDD from 5.6 to 18.7 ps/nm. The tunability of frequency difference and sub-pulse separation may arise from the group-delay-related filtering effect, as discussed detailly in Supplementary Note 5. The group delay differences for aforementioned operations range from 0 to ~1 ps, indicating a remarkable tolerance of the synchronized dual-wavelength mode-locked lasers.

**Fig. 3:**
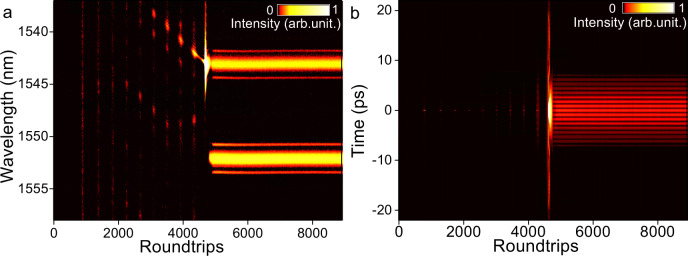
Buildup dynamics of the synchronized dual-wavelength mode-locked solitons. **a** Spectra and **b** field autocorrelation traces.

By means of the time-stretch dispersive Fourier transform technique^39–41^, we reveal the buildup process of synchronized dual-wavelength mode-locked solitons. As illustrated in Fig. 3a, the lasing wave experiences relaxation oscillation, beating dynamics and finally evolves into the synchronized dual-wavelength mode-locked state at the ~4930th roundtrip. At the steady state, the spectral sidebands are clearly observed on the waveform, confirming the reliability of the single-shot spectral measuring system. Note that the two spectra synchronously reach steady states and the field autocorrelation traces in Fig. 3b displays stable multi-peak structure, confirming the synchronized mode-locking nature. The buildup dynamics of synchronized three-wavelength mode-locked solitons are shown in Supplementary Fig. 6a, b, where the laser evolves analogically with that in Fig. 3 before reaching the self-consistent state.

Further increasing the pump power to 71 mW, restricted by the effective gain bandwidth^42^, the output power almost enlarges linearly while the single-pulse evolves into two- and triple-pulses sequently (Supplementary Note 7). In this case, the maximum output power is 1.44 mW, and the single-pulse energy spans from ~30 to ~70 pJ. Note that for the two- and triple-pulse operations, multi-peak structure is always observed on the AC trace and retrieved pulse profile, which also reflects the synchronized property of the multiple pulses.

**Fig. 4:**
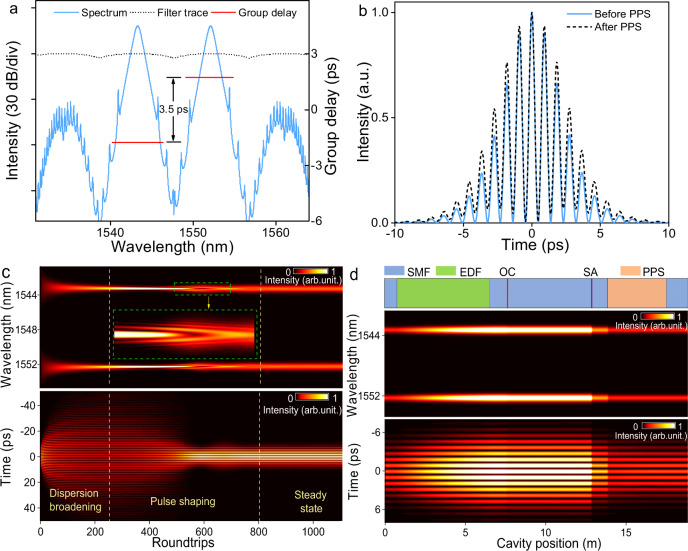
Simulation results of synchronized dual-wavelength mode-locked solitons. **a** Spectrum (slight blue), filter curve (black dashed), and group delay (red). **b** Profile of solitons (the slight blue solid and black dashed curves correspond to pulses before and after the PPS). **c**, **d** Spectral (upper panel) and temporal (lower panel) evolutions of soliton versus roundtrips and cavity position, respectively.

Based on the lumped propagation model in which each cavity component is modeled by a transmission function^7,43^, we simulate the establishment dynamics of the synchronized dual-wavelength mode-locked solitons (see “Methods”). The pulse propagation in fiber is modeled by the generalized nonlinear Schrodinger equation and solved with the typical split-step Fourier technique^44^. When only spectral filtering (black dashed curve in Fig. 4a) is considered, the two pulses propagate at different velocities, similar with the results in ref. ^16^. After introducing a group delay difference of 3.5 ps (comparable with ~3.25 ps in the experiment) to compensate that of the cavity (red solid curve in Fig. 4a), a typical synchronized dual-wavelength mode-locking state is automatically established in the fiber laser with the assistance of the saturable absorber, despite the residual group delay difference of ~0.07 ps. Interestingly, the final state of the laser is independent of the initial input noise pulse, i.e., the similar operation can be achieved by using dual-wavelength noise pulses centered at 1543.3 and 1552.1 nm (Fig. 4) or single-wavelength noise pulse centered at 1547.8 nm (Supplementary Note 8).

The simulated soliton in Fig. 4a, b excellently agrees with that of the experiments in Fig. 2. For example, the 3-dB bandwidth and duration of the pulse envelope are 0.54 nm and 5 ps, respectively, which give a time-bandwidth product of ~0.33, further validating the chirp-free property of the soliton. The dynamic evolutions of such soliton versus roundtrips and cavity positions are shown in Fig. 4c and d, respectively. The simulation starts from dual-wavelength low-intensity noise pulses, and they gradually evolve into the synchronized mode-locked state after ~800 roundtrips, which coincide with the buildup dynamics shown in Fig. 3. In the first stage, the spectral locations of the initial pulses are roughly confined by the filter, and periodically modulated fine structure appears in the time domain due to the interference of two-wavelength solitons. Then, the positions of peak wavelengths become stable after ~200 roundtrips. Correspondingly, the pulse packet broadens quickly to the maximum value under the strong group velocity dispersion. After that, with the assistance of the saturable absorber, the envelope of the pulse starts to narrow and reaches its minimum at the ~600th roundtrip. At the same time, the intensity of pulse increases and new spectral components (inset of Fig. 4c) appear due to the self-phase modulation effect. Eventually, the pulse evolves into a self-consistent state after the pulse shaping process. It is indicated that the group delay compensation dominate the synchronization of wavelength-different pulses, under the well-known balances of gain by the saturable absorption and anomalous-dispersion by self-phase modulation that result in the formation of solitons^45^.

In a single circulation, as depicted in Fig. 4d, the laser propagates in the following order: WDM *→* EDF *→* OC *→* PI-ISO *→* CNT-SA *→* PPS. As the PPS applies a large amount of GDD at a small section of fiber in the cavity, the pulse packet disperses abruptly with the slight degeneration of the modulation depth, as compared in Fig. 4b. Simultaneously, the spectra are confined at two fixed wavelengths due to the spectral filtering effect. After that, the pulse propagates through the residual single mode fiber (SMF) and enters the WDM for the next circulation. Apart from a clear change at the PPS, the profile and modulation period of the pulse packet change slightly inside the cavity, which is somewhat similar with that of single-wavelength fundamental solitons^46^. In the experiment, we found that the pulses at different cavity positions exhibited the similar physical properties, which further validated the reliability of the simulation results.

### Synchronized multi-wavelength mode-locked soliton fiber lasers

**Fig. 5:**
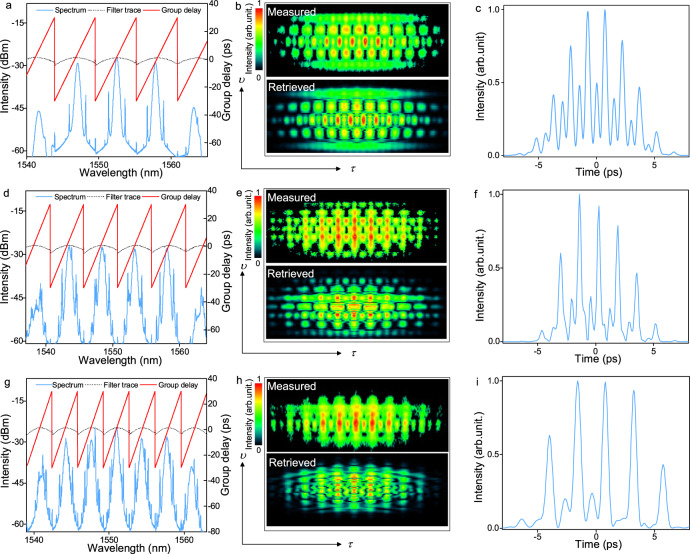
Synchronized three- to five-wavelength mode-locking of soliton fiber lasers. Spectra, FROG spectrograms, and retrieved pulse profiles of **a**–**c** three-, **d**–**f** four-, and **g**–**i** five-wavelength mode-locked solitons, respectively.

By finely optimizing the group delay function and the bandwidth of PPS, we obtain three- to five-wavelength mode-locked solitons, as shown in Fig. 5. During the experiment, the signal-to-noise ratio of the pulse decreases from ~55 dB to ~40 dB when the number of the spectrum increases from two to five, indicating the slight degeneration of the laser stability. It is worth noting that all mode-locking operations are self-started and almost keep unchanged during the whole experiment. Attributing to the gain competition effect, the adjacent spectrum should be well separated to form a soliton in the fiber laser. Thus, the limiting factor for the number of the total spectra is the gain bandwidth of the EDF.

Taking four-wavelength solitons in Fig. 5d as an example, the spectra are equally spaced at 1543.5, 1548.4, 1553.3, and 1558.2 nm, and the intensity difference among them is less than 3 dB. The FROG spectrograms are illustrated in Fig. 5e, which give the retrieved temporal profile with the sub-pulse duration of 0.34 ps (Fig. 5f), comparable with that of dual-wavelength soliton (0.31 ps). One can find that the weak sub-pulses are suppressed with the increase of wavelength number, which results in a reduction of duty ratio and coincides with the mode-locking principle.

## Discussions

The synchronized multi-wavelength mode-locking of fiber lasers also follows from the minimum loss principle^47^, in which the pulse strives to find the complex temporal structure that minimizes loss during the propagation in cavity. Specifically, the formation of such unique wavepackets relies on three key factors. First, the net group delay difference of cavity should be trimmed to a small range to realize synchronization of the multi-wavelength solitons. For example, in the present settings, the net group delay difference of the cavity should be confined to ~1 ps to achieve synchronized mode-locking states. Second, despite the existence of residual cavity dispersion, the saturable absorber forces solitons at different wavelengths to synchronize as its loss is inversely related to the pulse intensity. Further, when the multi-peak structure of wavepackets is formed, the saturable absorber suppresses the background noise due to the saturable nonlinear loss, which also stabilizes the synchronizing between multi-wavelength soliton pulses. The third is the group-delay-related filtering effect that results in the multi-wavelength lasers. Actually, for group delay with small amplitudes (<10 ps), the spectral filtering effect becomes ignorable and the discontinuity of group delay function should be particularly considered for future studies.

To confirm the aforementioned principle, we have demonstrated the other approach to realize synchronized mode-locking by introducing spectral filtering effect in near-zero dispersion fiber lasers. As shown in Supplementary Fig. 9, except the elimination of spectral sidebands, the laser properties are similar with the synchronized dual-wavelength mode-locked solitons in Fig. 2a. However, due to the gain competition in the near-zero-dispersion regime, such scheme is difficult to support multi-wavelength synchronized solitons and the output laser pulses are less stable than that based on PPS. Unlike gigahertz soliton formed in microresonator that each spectrum corresponds to single longitudinal mode^48^, the obtained terahertz wavepackets are composed of multi-wavelength synchronized soliton and each of soliton spectrum comprises ~10^5^ phase-locked longitudinal modes. The proposal opens an interesting direction in the study of laser mode-locking, and in the perspective of applications, the unique pulse sources can be applied in high-energy femtosecond pulse emission (Supplementary Note 10), difference-frequency terahertz waves generation, and nonlinear field-enhancement Raman microscopy.

## Methods

### Experiment setup

The erbium-doped fiber laser is composed of a wavelength division multiplexer (WDM), 6-m EDF (dispersion parameter *D*: 16.7 ps nm^−1^ km^−1^, EDFC-980-HP), a 90:10 output coupler (OC), a PPS, a CNT-SA, and a PI-ISO. The pigtails of fiber components are standard SMFs (*D*: 17 ps nm^−1^ km^−1^, SMF 28e+) with the total length of 9.2 m. The PPS has 3.7-m optical path with an innate GDD of 0.35 ps nm^−1^. Based on aforementioned parameters, the total length and net group velocity dispersion are estimated as 18.9 m and −0.52 ps^2^, respectively.

The PPS consists of a diffraction grating, a parabolic mirror, a liquid crystal on silicon spatial light modulator (SLM). As shown in Fig. 1b, the incident light is collimated by L1, angularly dispersed by the grating, and then reflected onto the SLM with a cylindrical mirror. By varying the pixel values of the SLM, it is possible to change the phase and amplitude of the diffracted spectral components. After that, the cylindrical mirror directs them onto the same grating, and L2 focuses the diffracted beam into the output port of the PPS. The reflectivity of SLM depends on the group delay value, which introduces a periodically modulated spectral filtering effect^34^.

### Numerical simulations

As shown in Eq. (1), the group velocity dispersion, Kerr nonlinearity, gain, and loss of each section of fibers are involved in the simulation. Besides this, we specially take into account the group delay and spectral filtering effect induced by the PPS, as shown in Fig. 4a. The variables *z, t*, and *u*(*z,t*) correspond to the propagate coordinate, the pulse local time, and slow varying wavepacket, respectively. *k*_2_ is the second-order dispersion coefficient, *γ* the cubic refractive nonlinearity of the fiber, and Ω_*g*_ the gain bandwidth of the EDF. *α* represents the loss of the optical fiber, and *g* = *g*_0_exp(−*E*_p_/*E*_s_) is the saturated gain of EDF, where *g*_0_, *E*_p_, and *E*_s_ are the small-signal gain coefficient, pulse energy, and gain saturation energy respectively.

1 $$\begin{array}{c}\frac{\partial u(z,t)}{\partial z}=-i\frac{{k}_{2}}{2}\frac{{\partial }^{2}u(z,t)}{\partial {t}^{2}}+i\gamma {|u(z,t)|}^{2}\,u(z,t)+\frac{(g-\alpha )}{2}u(z,t)+\frac{g}{2{\varOmega^{2} }{g}}\frac{{\partial }^{2}u(z,t)}{\partial {t}^{2}}\,\end{array}$$

The generalized nonlinear Schrodinger equation is numerically solved with the typical symmetric split-step Fourier method^38^. The saturable absorber has a transmittance *T* = 0.46 − *T*_0_/[1 + *P*_(τ)_/*P*_sat_], where *T*_0_ is the modulation depth, *P*_(τ)_ is the instantaneous pulse power, and *P*_sat_ is the saturable power. The simulation parameters are consistent with their experimental values: *E*_s_ = 7.0 pJ, *T*_0_ = 0.06, *P*_sat_ = 10 W. For SMF, *g*_0_ = 0, *α* = 4.6 × 10^−5^ m^−1^, *k*_2_ = −0.0217 ps^2^ m^−1^, *γ* = 1.3 × 10^−3^ W^−1^ m^−1^. For PPS, the intrinsic dispersion parameter *k*_2_ = −0.1203 ps^2^ m^−1^, *γ* = 0. For EDF, *g*_0_ = 0.7 m^−1^, *k*_2_ = 0.0213 ps^2^ m^−1^, *γ* = 3.9 × 10^−3^ W^−1^ m^−1^.

In the simulation, the laser first propagates through 0.8-m pigtail of WDM, and then is amplified by the 6-m EDF. Due to the saturable amplification property of the EDF, the soliton almost increases linearly in the forepart while enlarges slowly in the rear part. After the EDF, the pulse passes through 0.8-m SMF and is outputted by the 10% optical coupler. Then, it further propagates through 5.2-m SMF and a CNT-SA, in which the duration and intensity decrease due to the saturable absorption effect. At the position of 13.8 m, the PPS imparts the GDD and spectral filtering to the pulse.

### Measurement system

An OSA (YOKOGAWA, AQ6370), a FROG (FEMTOEASY, FS-600), an intensity autocorrelator (Pulsecheck, USB-150), and a radiofrequency analyzer (Agilent, E4440A) are utilized to measure the optical spectrum, pulse profile, pulse duration, and signal-to-noise ratio, respectively. The spectral dynamics are real-time captured by the DFT system composed of a 7-km-long dispersion-compensating fiber (DCF: YOFC, G652 DCF-C) with the dispersion of −150 ps nm^−1^ km^−1^, a 5-GHz photodetector (THORLABS, DET09CFC/M) together with a 4 GHz real-time oscilloscope (Lecroy, 740Zi-A). The electronic-based spectral resolution of the system is calculated as 0.24 nm according to the overall limitation of DFT on the spectral resolution^39^.

### Reporting summary

Further information on research design is available in the Nature Research Reporting Summary linked to this article.

## Supplementary information

Supplementary Information

Reporting Summary

## Data Availability

The data supporting the results of this paper are available from the corresponding author upon reasonable request.

## Code availability

The simulation code is available upon reasonable written request, which excludes any commercial interest.
