# Supplementary material for: Synchronized multi-wavelength soliton fiber laser via intracavity group delay modulation
Source: Nat Commun. 2021 Nov 18;12:6712. doi: 10.1038/s41467-021-26872-x (PMC8602375; doi:10.1038/s41467-021-26872-x)
Supplement: Supplementary file 1 — Supplementary Information [file 41467_2021_26872_MOESM1_ESM.pdf]

Supplementary Information:

Synchronized multi-wavelength soliton fiber laser via intracavity group delay modulation

Dong Mao[1], Huaqiang Wang[1], Heze Zhang[1], Chao Zeng[1], Yueqing Du[1], Zhiwen He[1], Zhipei Sun[2,*] & Jianlin Zhao[1,*]

[1]*MOE Key Laboratory of Material Physics and Chemistry under Extraordinary Conditions, and Shaanxi Key Laboratory of Optical Information Technology, School of Physical Science and Technology, Northwestern Polytechnical University, Xi'an 710129, China*

[2]*Department of Electronics and Nanoengineering and QTF Centre of Excellence, Aalto University, Aalto, Finland*

[*]Corresponding author: zhipei.sun@aalto.fi; jlzhao@nwpu.edu.cn

Contents

Supplementary Note 1 - Performances of mode-locked solitons before imparting group delay....................... S3

Supplementary Note 2 - Radio frequency spectrum at 1 GHz span and autocorrelation trace at 150 ps span of synchronized dual-wavelength mode-locked solitons... S3

Supplementary Note 3 - Scalar and vector synchronized dual-wavelength mode-locked solitons S4

Supplementary Note 4 - Soliton properties for each spectrum of dual-wavelength solitons............................ S5

Supplementary Note 5 - Four typical synchronized dual-wavelength mode-locked solitons S6

Supplementary Note 6 - Buildup process of synchronized three-wavelength mode-locked solitons............... S8

Supplementary Note 7 - Evolution of average power and pulse energy versus pump power S9

Supplementary Note 8 - Simulated synchronized dual-wavelength mode-locked solitons initiated from the single-wavelength noise pulse.. S9

Supplementary Note 9 - Synchronized dual-wavelength mode-locking in near-zero-dispersion fiber lasers. S11

Supplementary Note 10 - Nonlinear propagation of synchronized dual-wavelength mode-locked solitons... S11

References S12

Supplementary Note 1 - Performances of mode-locked solitons before imparting group delay

The fiber laser operates at the standard single-wavelength mode-locked state before imparting group delay with the programmable pulse shaper (PPS), as shown in Supplementary Fig. 1a-b. The output spectrum spans from 1540 nm to 1560 nm with pairs of sharp sidebands, confirming that the fiber laser operates at the anomalous-dispersion regime[1]. The bandwidth and duration of the pulses are 2.9 nm and 1.12 ps, which give a time-bandwidth product of 0.43, indicating a chirp-free soliton. The asymmetrical sidebands on the spectrum may result from the uneven gain spectrum of the medium[2] and the third-order dispersion of fiber laser[3].

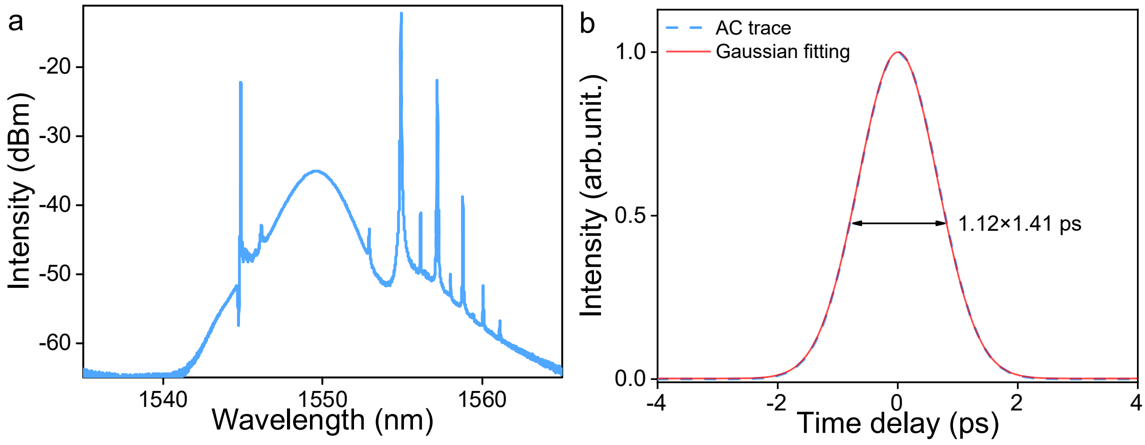

Supplementary Figure 1 | Performances of mode-locked soliton before imparting GD. a, Spectrum, b, autocorrelation (AC) trace and the Gaussian fitting.

Supplementary Note 2 - Radio frequency spectrum at 1 GHz span and autocorrelation trace at 150 ps span of synchronized dual-wavelength mode-locked solitons

The beating frequencies of synchronized dual-wavelength solitons range from ~0.4 to ~1.26 THz, which are much higher than the bandwidth of our available photodetector and radio frequency analyzer (usually less than 0.1 THz). At the 1 GHz span, there is no other strong intensity modulation on the radio frequency analyzer (Supplementary Fig. 2a), indicating the good stability of the synchronized

dual-wavelength operation. Furthermore, only single wavepacket is observed on the autocorrelator at the maximum span of 150 ps and the clear interference fringe is observed (Supplementary Fig. 2b), indicating the dual-wavelength pulses are synchronized in time domain.

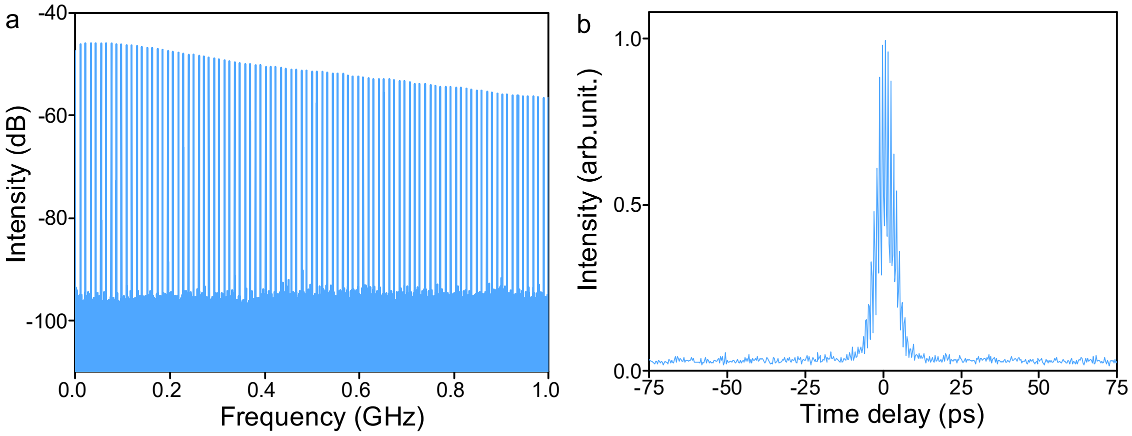

Supplementary Figure 2 | Properties of synchronized dual-wavelength mode-locked solitons. a, RF spectrum, b, AC trace.

Supplementary Note 3 - Scalar and vector synchronized dual-wavelength mode-locked solitons

The polarization states of synchronized dual-wavelength mode-locked lasers are further analyzed by a polarization beam splitter, and each component is measured by the optical spectrum analyzer and intensity autocorrelator. For most cases, the scalar dual-wavelength solitons are obtained from the fiber laser, in which the two spectra have the same polarization state (Supplementary Fig. 3a-b) and ultrahigh-repetition-rate sub-pulses always can be observed, as displayed in Fig. 2d-e in the main text. However, at special settings of polarization controller, the vector synchronized dual-wavelength mode-locked solitons can also be achieved. In this case, the two spectra are orthogonally-polarized and the attendant spectra are eliminated, as plotted in Supplementary Fig. 3c. The multi-peak fine structure disappears as each spectrum corresponds to an orthogonally-polarized component of pulse, as shown in the Supplementary Fig. 3d.

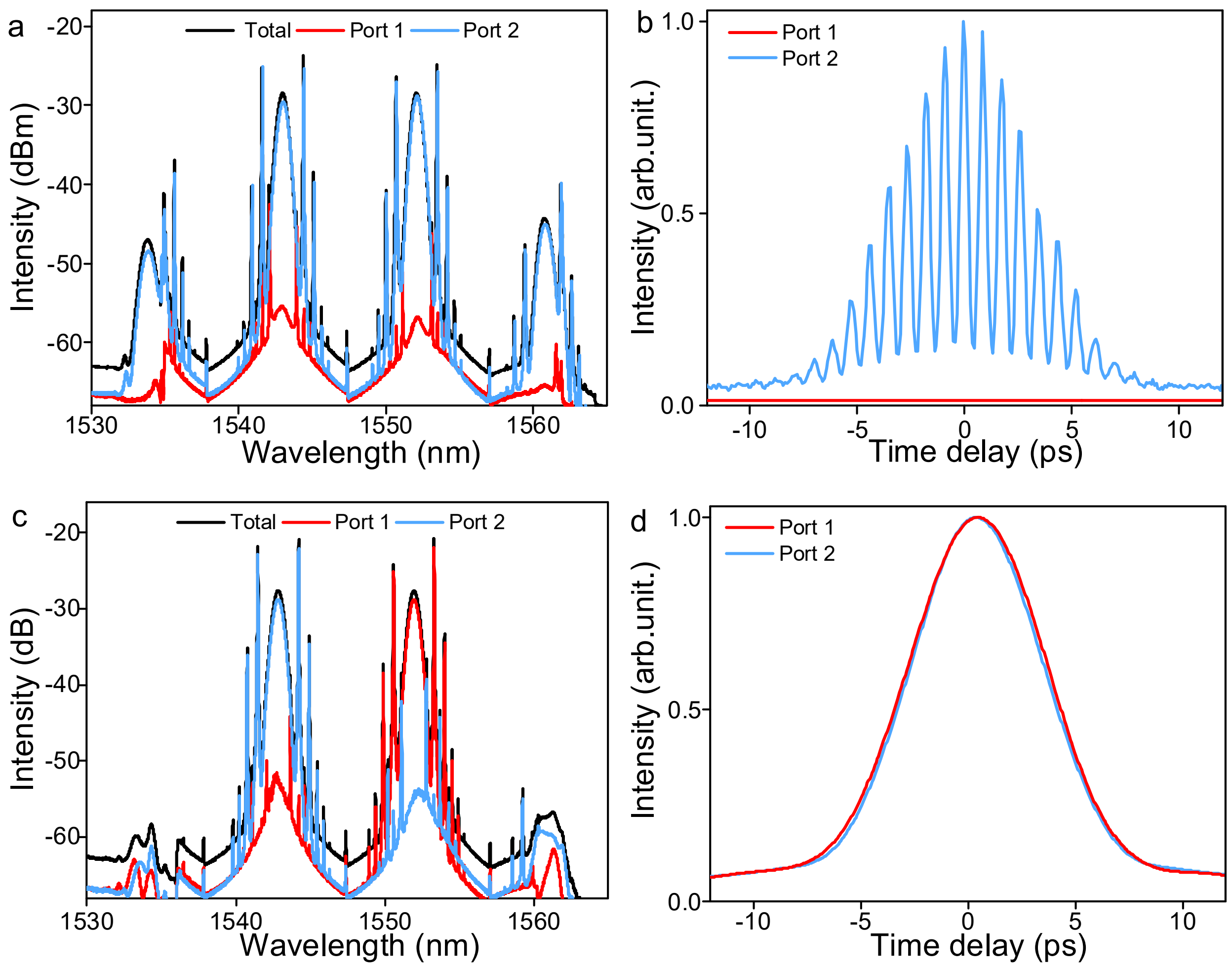

Supplementary Figure 3 | a, b Scalar and c, d vector synchronized dual-wavelength mode-locked solitons. a, c, Spectra, and b, d, AC traces of the two orthogonally-polarized components.

Supplementary Note 4 - Soliton properties for each spectrum of dual-wavelength solitons

We further study the dual-wavelength solitons by filtering out each spectrum external to the cavity with a fiber Bragg grating and a circulator. As shown in Supplementary Fig. 4a-b, the two pulses centered at 1543 nm and 1552 nm have durations of ~4.89 and ~5.5 ps respectively, which is consistent with that of the pulse envelope in Figs. 2d-e of the main text. Our results confirm that, the envelope width of the synchronized multi-wavelength mode-locked pulse rests with the soliton duration of each spectral component formed in the anomalous-dispersion regime.

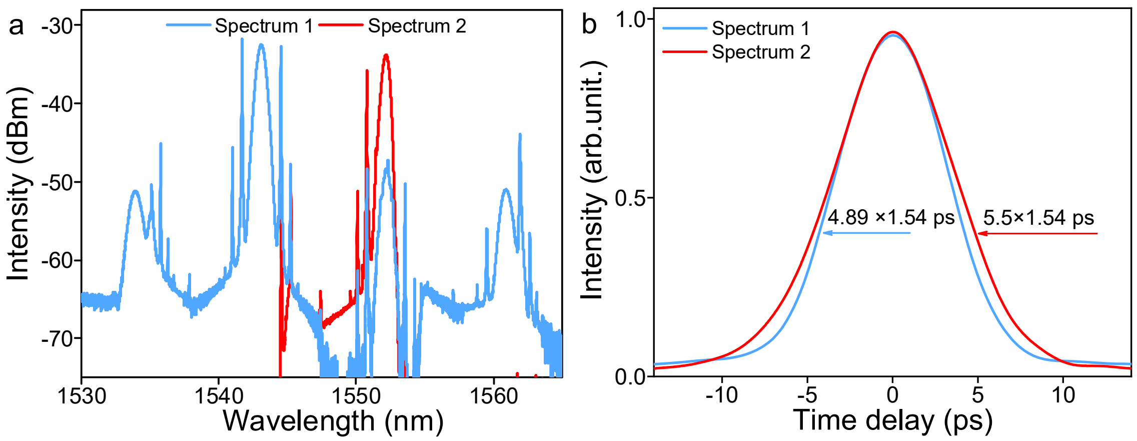

Supplementary Figure 4 | Performance of dual-wavelength solitons after filtering out each spectrum. a, Spectra, and b, AC traces of the filtered pulses.

Supplementary Note 5 - Four typical synchronized dual-wavelength mode-locked solitons

The performance of synchronized dual-wavelength solitons depends on the bandwidth and group delay imparted by the PPS. Supplementary Fig. 5 shows four typical synchronized dual-wavelength mode-locked solitons for group delay dispersion (GDD) of 5.6 ps/nm, 7.4 ps/nm, 9.4 ps/nm, and 18.7 ps/nm respectively. It is demonstrated that the frequency difference of two spectra, equivalent to the repetition rate of sub-pulses, diminishes inversely with the increase of the GDD. Simultaneously, the widths of each spectrum and the pulse envelope change slightly due to the spectral filtering effect. The group delay differences for aforementioned operations range from 0 ps to ~1 ps, indicating a remarkable tolerance of synchronized dual-wavelength mode-locked lasers.

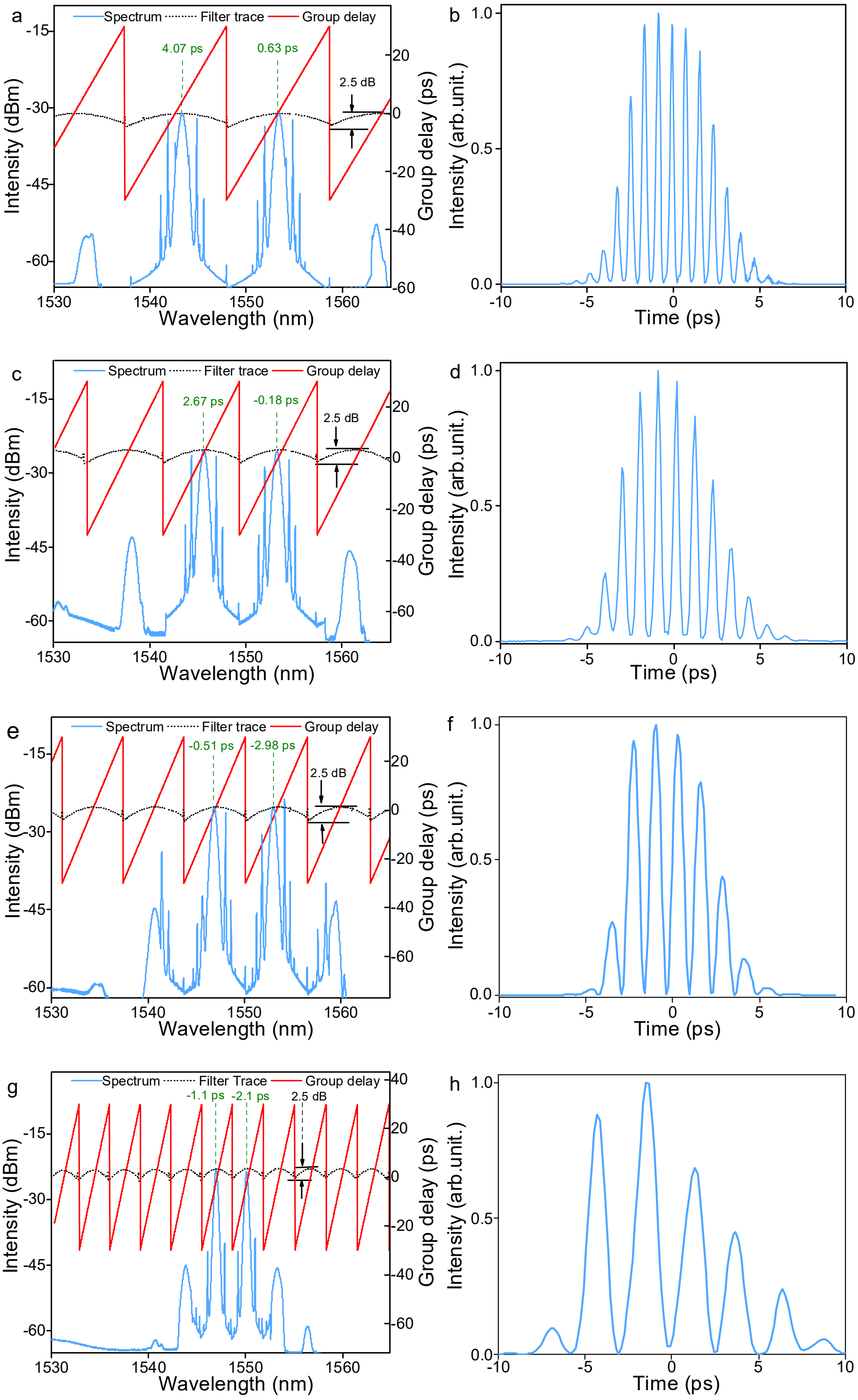

Supplementary Figure 5 | Performance of four typical synchronized dual-wavelength mode-locked solitons. The GDDs for a-b, c-d, e-f and g-h are 5.6 ps/nm, 7.4 ps/nm, 9.4 ps/nm, and 18.7 ps/nm respectively. a, c, e, g, Spectra

(slight blue), filter curves (black dashed) and group delays (red). b, d, f, h, Retrieved pulse profiles.

Supplementary Note 6 - Buildup process of synchronized three-wavelength mode-locked solitons

We record the buildup process of synchronized three-wavelength mode-locked solitons by the time-stretch dispersive Fourier transform (DFT) technique, as shown in Supplementary Fig. 6. After propagating through 7000-m dispersion compensation fiber (YOFC, G652 DCF-C), the spectral information of the pulse can be mapped into the temporal domain[4-6]. Then a 5 GHz high-speed photodetector (THORLABS, DET09CFC/M) and a 4 GHz real time oscilloscope (Lecroy, 740Zi-A) are utilized to capture and record the processed signal. Based on the overall limitation of DFT[4], the spectral resolution of this real-time spectral measuring system is calculated as 0.24 nm. Analogous to the results in Fig. 3 of the manuscript, the lasing wave experiences relaxation oscillation, beating dynamics and finally achieves the three-wavelength matryoshka mode-locked state at the ~5100th roundtrip. The three spectral components synchronously reach steady state and the field autocorrelation trace displays stable multi-peak structure, indicating the synchronized mode-locking nature.

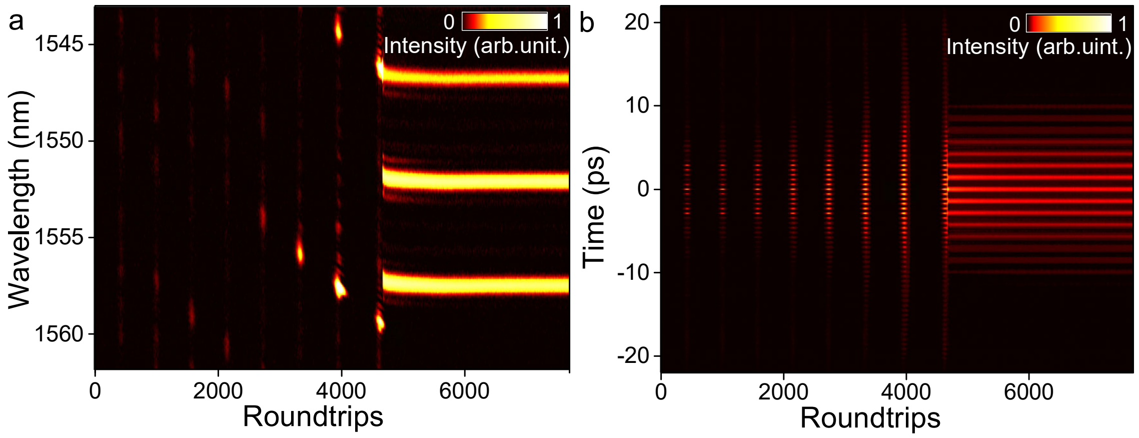

Supplementary Figure 6 | Buildup process of synchronized three-wavelength mode-locked solitons. a, Spectral characterization, and b, field autocorrelation.

Supplementary Note 7 - Evolution of average power and pulse energy versus pump power

By enhancing the pump power from 22 mW to 71 mW, the average output power almost enlarges linearly from 0.32 mW to 1.44 mW. Simultaneously, the laser operation evolves from single- to two- and triple-pulses states and the pulse energy follows a quasi-periodical behavior[7], as shown in Supplementary Fig. 7. During this process, the retrieved pulses always display stable multi-peak structure with the fixed temporal separation, which reflects that the two pulses are synchronized during the whole experiment. By using a coupler with 40% output ratio, the output power reaches ~3.9 mW. The output power may be further enhanced by increasing the pump strength or using large-mode-area fiber laser.

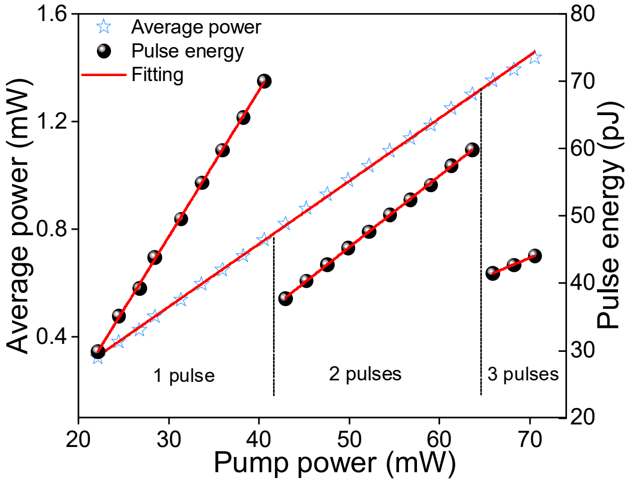

Supplementary Figure 7 | Pulse evolution versus pump power. Single pulse (22 mW~41 mW); two pulses (42 mW~64 mW); triple pulses (65 mW~ 71 mW).

Supplementary Note 8 - Simulated synchronized dual-wavelength mode-locked solitons initiated from the single-wavelength noise pulse

The final state of the fiber laser is independent of initial conditions, for example, similar synchronized dual-wavelength mode-locked solitons can be achieved by using dual-wavelength noise pulses

centered at 1543.3 and 1552.1 nm (Fig. 4 of the main text) or single-wavelength noise pulse centered at 1547.8 nm (Supplementary Figure 8). The corresponding time bandwidth product is calculated as 0.35, which indicates the chirp-free nature of the soliton. Our results confirm that, despite the existence of residual cavity GDD, the fiber laser can evolve from different initial conditions to the similar synchronized dual-wavelength mode-locking state due to the saturable absorption effect.

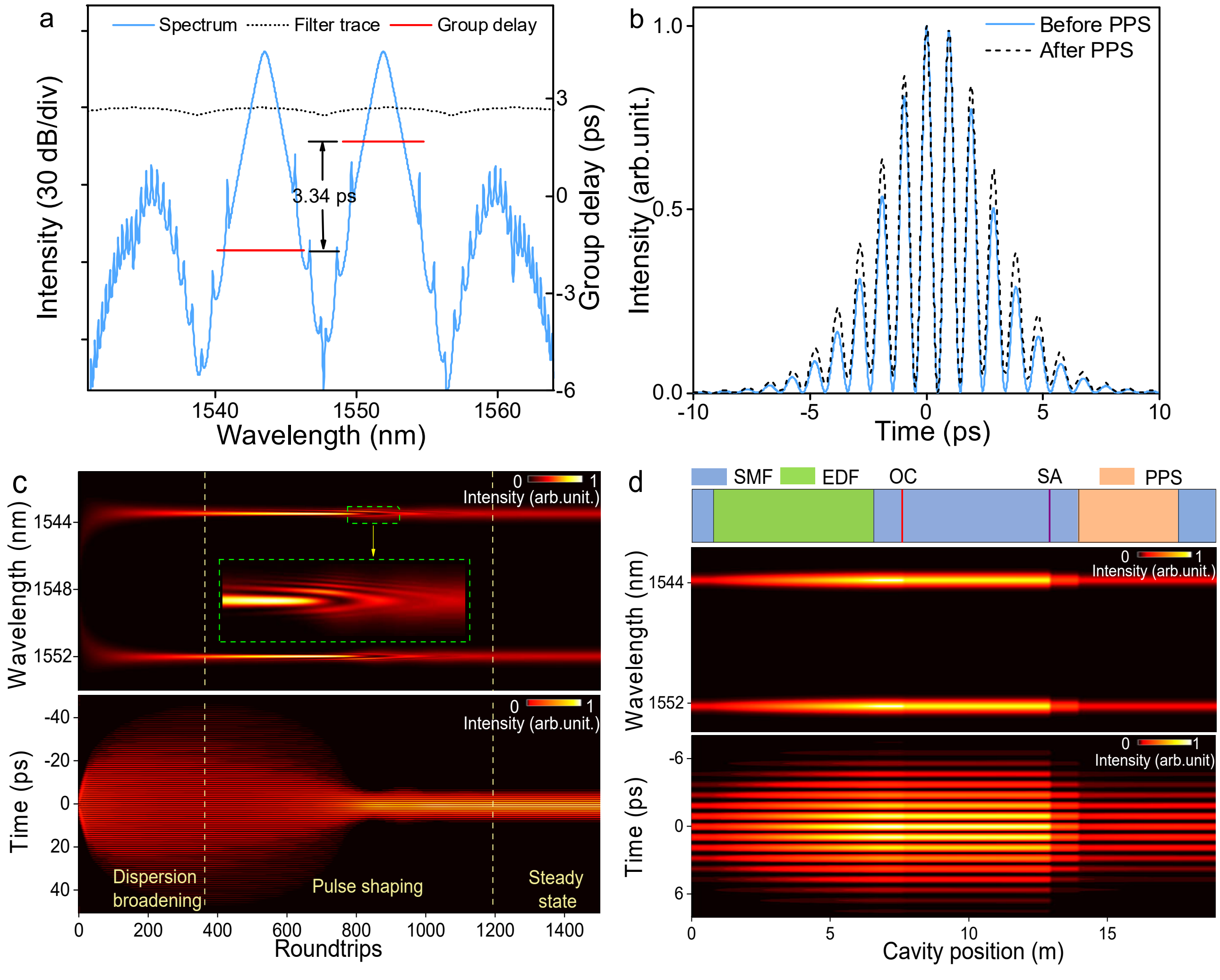

Supplementary Figure 8 | Simulation results of synchronized dual-wavelength mode-locked solitons initiated from the single-wavelength noise pulse. a, Spectrum (slight blue), filter curve (black dashed) and group delay (red). b, Profile of solitons (the slight blue solid and black dashed curves correspond to pulses before and after the PPS). c, d, Spectral (upper panel) and temporal (lower panel) evolutions of soliton versus roundtrips and cavity position, respectively.

Supplementary Note 9 - Synchronized dual-wavelength mode-locking in near-zero-dispersion fiber lasers

By managing the cavity dispersion into the near-zero dispersion regime and replacing the PPS with a fiber filter based on inter-mode interference, another type of synchronized dual-wavelength mode-locked solitons can be also achieved. The optical spectrum and pulse profile are illustrated in Supplementary Figs. 9a and 9b, respectively. One can observe that, except the elimination of spectral sidebands, the laser properties are similar with the synchronized dual-wavelength mode-locked solitons in Fig. 2 of the main text. However, due to the gain competition in the near-zero-dispersion regime, such scheme is difficult to support multi-wavelength synchronized solitons and the output laser pulses are less stable than that based on PPS. This result confirms that synchronized dual-wavelength mode-locking is a ubiquitous phenomenon in fiber lasers, and other types of operation may be achieved via engineering the cavity design or managing the group delay and filtering effects.

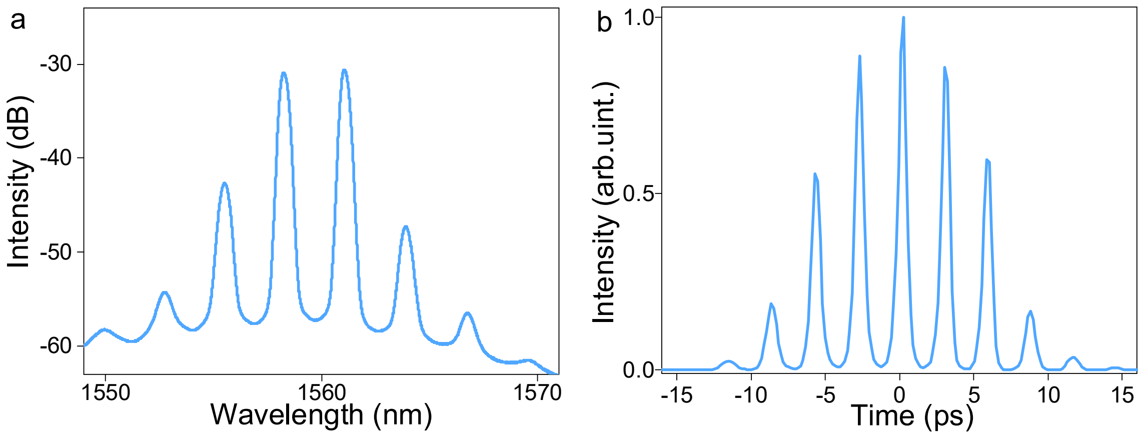

Supplementary Figure 9 | Performance of synchronized dual-wavelength mode-locked fiber lasers based on spectral filtering effect in near-zero dispersion regimes. a, Spectrum, and b, retrieved pulse profile.

Supplementary Note 10 - Nonlinear propagation of synchronized dual-wavelength mode-locked solitons

The average power of synchronized dual-wavelength mode-locked solitons can be amplified to 21 mW

(corresponding to the pulse energy of 1.9 nJ) with a home-made erbium-doped fiber amplifier. Such unique laser sources can be used to explore the nonlinear phenomena in single-/few-/multi-mode fibers and nonlinear crystals. Supplementary Fig. 10 shows the properties of the dual-wavelength soliton after propagating through 500-m single-mode fiber. Attributed to the modulation instability and other nonlinear effects, the multi-peak wavepackets gradually evolves to a 438 fs pulse.

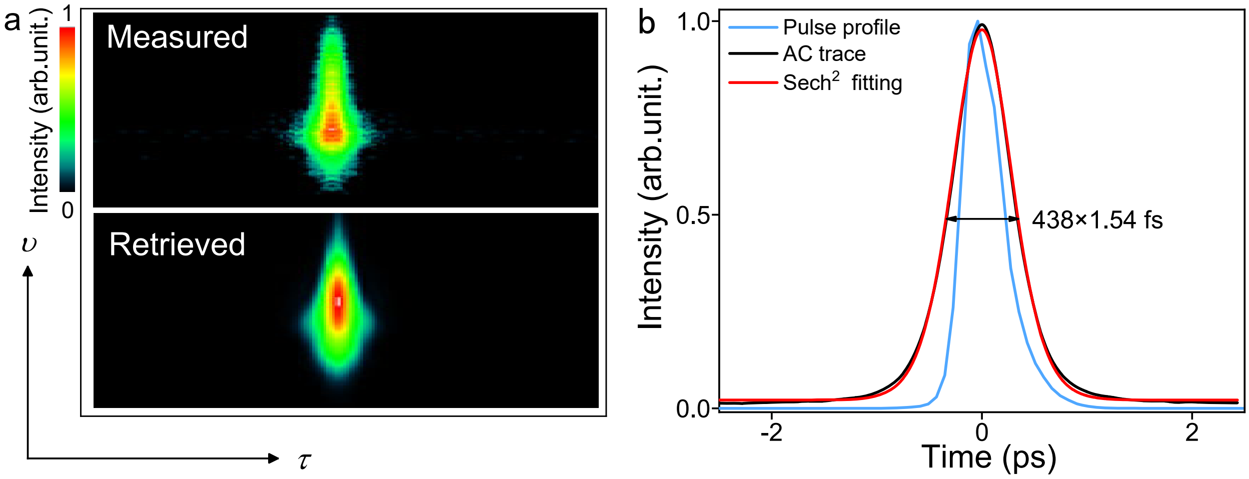

Supplementary Figure 10 | Propagation property of synchronized dual-wavelength mode-locked soliton with 500-m single-mode fiber. a, FROG trace, b, retrieved pulse profile and AC trace.

References

1 Nelson, L. E., Jones, D. J., Tamura, K., Haus, H. A. & Ippen, E. P. Ultrashort-pulse fiber ring lasers. *Appl. Phys. B* **65**, 277-294 (1997).

2 He, W., Pang, M., Menyuk, C. R. & Russell, P. S. Sub-100-fs 1.87 GHz mode-locked fiber laser using stretched-soliton effects. *Optica* **3**, 1366-1372 (2016).

3 Logvin, Y., Kalosha, V. P. & Anis, H. Third-order dispersion impact on mode-locking regimes of Yb-doped fiber laser with photonic bandgap fiber for dispersion compensation. *Opt. Express* **15**, 985-991 (2007).

4 Goda, K. & Jalali, B. Dispersive Fourier transformation for fast continuous single-shot measurements. *Nat. Photon.* **7**, 102-112 (2013).

5 Herink, G., Jalali, B., Ropers, C. & Solli, D. R. Resolving the build-up of femtosecond mode-locking with single-shot spectroscopy at 90 MHz frame rate. *Nat. Photon.* **10**, 321-326 (2016).

6 Ryczkowski, P. *et al.* Real-time full-field characterization of transient dissipative soliton dynamics in a mode-locked laser. *Nat. Photon.* **12**, 221-227 (2018).

7 Tang, D. Y., Zhao, L. M., Zhao, B. & Liu, A. Q. Mechanism of multisoliton formation and soliton energy

quantization in passively mode-locked fiber lasers. *Phys. Rev. A* **72**, 9 (2005).
