# Supplementary material for: Synchronized multi-wavelength soliton fiber laser via intracavity group delay modulation
Source: Nat Commun. 2021 Nov 18;12:6712. doi: 10.1038/s41467-021-26872-x (PMC8602375; doi:10.1038/s41467-021-26872-x)
Supplement: Supplementary file 2 — Reporting Summary [file 41467_2021_26872_MOESM2_ESM.pdf]

Corresponding author(s): Zhipei Sun NCOMMS-21-14361A

Lasing Reporting Summary

Nature Research wishes to improve the reproducibility of the work that we publish. This form is intended for publication with all accepted papers reporting claims of lasing and provides structure for consistency and transparency in reporting. Some list items might not apply to an individual manuscript, but all fields must be completed for clarity.

For further information on Nature Research policies, including our data availability policy, see Authors & Referees.

Experimental design

Please check: are the following details reported in the manuscript?

1. Threshold

Plots of device output power versus pump power over a wide range of values indicating a clear threshold

☐ Yes ☒ No

The work in the manuscript do not need such a threshold.

2. Linewidth narrowing

Plots of spectral power density for the emission at pump powers below, around, and above the lasing threshold, indicating a clear linewidth narrowing at threshold

☐ Yes ☒ No

The work in the manuscript do not need to plot spectral power density.

Resolution of the spectrometer used to make spectral measurements

☒ Yes ☐ No

Measurement system in the manuscript.

3. Coherent emission

Measurements of the coherence and/or polarization of the emission

☐ Yes ☒ No

The work in the manuscript do not need to measure the coherence and polarization of the emission.

4. Beam spatial profile

Image and/or measurement of the spatial shape and profile of the emission, showing a well-defined beam above threshold

☐ Yes ☒ No

Single-mode fiber lasers only have Gaussian profile of the emission in spatial domain.

5. Operating conditions

Description of the laser and pumping conditions
Continuous-wave, pulsed, temperature of operation

☒ Yes ☐ No

Experimental setup in the manuscript.

Threshold values provided as density values (e.g. W cm^{-2} or J cm^{-2}) taking into account the area of the device

☐ Yes ☒ No

The work in the manuscript do not need such threshold values.

6. Alternative explanations

Reasoning as to why alternative explanations have been ruled out as responsible for the emission characteristics
e.g. amplified spontaneous, directional scattering; modification of fluorescence spectrum by the cavity

☐ Yes ☒ No

The laser operates at mode-locked state by a saturable absorber, emitting ultrashort pulse.

7. Theoretical analysis

Theoretical analysis that ensures that the experimental values measured are realistic and reasonable
e.g. laser threshold, linewidth, cavity gain-loss, efficiency

☒ Yes ☐ No

Experimental setup in the manuscript.

8. Statistics

Number of devices fabricated and tested

☒ Yes ☐ No

Measurement system in the manuscript.

Statistical analysis of the device performance and lifetime (time to failure)

☐ Yes ☒ No

The work in the manuscript do not need the statistical analysis of the device performance and lifetime.
